# Supplementary material for: The YTHDF proteins ECT2 and ECT3 bind largely overlapping target sets and influence target mRNA abundance, not alternative polyadenylation
Source: eLife. 2021 Sep 30;10:e72377. doi: 10.7554/eLife.72377 (PMC8789314; doi:10.7554/eLife.72377)
Supplement: Figure 5—source data 1. [file elife-72377-fig5-data1.zip › ECT2-3_Targets_v2_Figure5-Source_data_1.pdf]

Figure 5—SourceData1

Figure panel

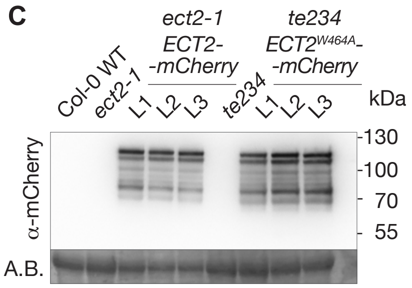

α-mCherry western blot
(chemiluminescence)

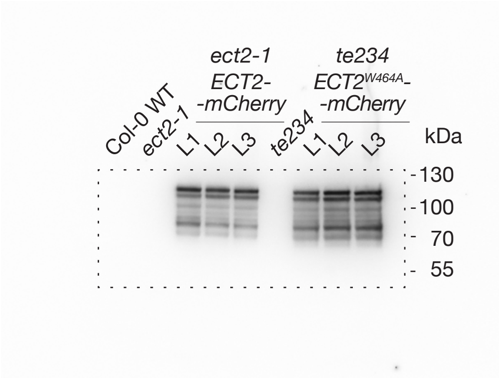

Source Data 2

Amido black staining

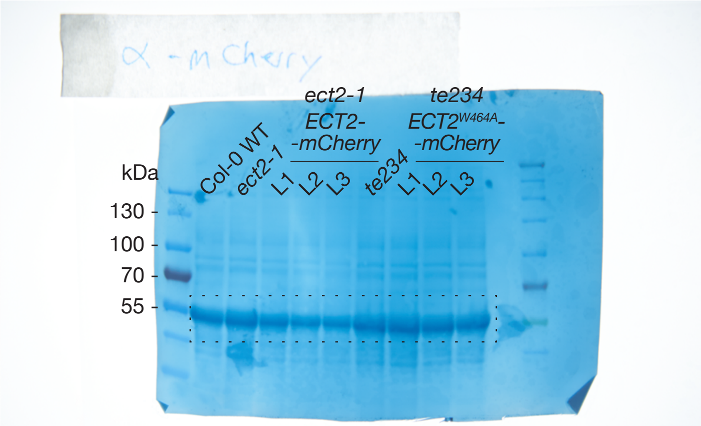

Source Data 3

Dotted outlines indicate the cropping applied to the figure
