# Supplementary figures and images for: The YTHDF proteins ECT2 and ECT3 bind largely overlapping target sets and influence target mRNA abundance, not alternative polyadenylation
Source: eLife. 2021 Sep 30;10:e72377. doi: 10.7554/eLife.72377 (PMC8789314; doi:10.7554/eLife.72377)

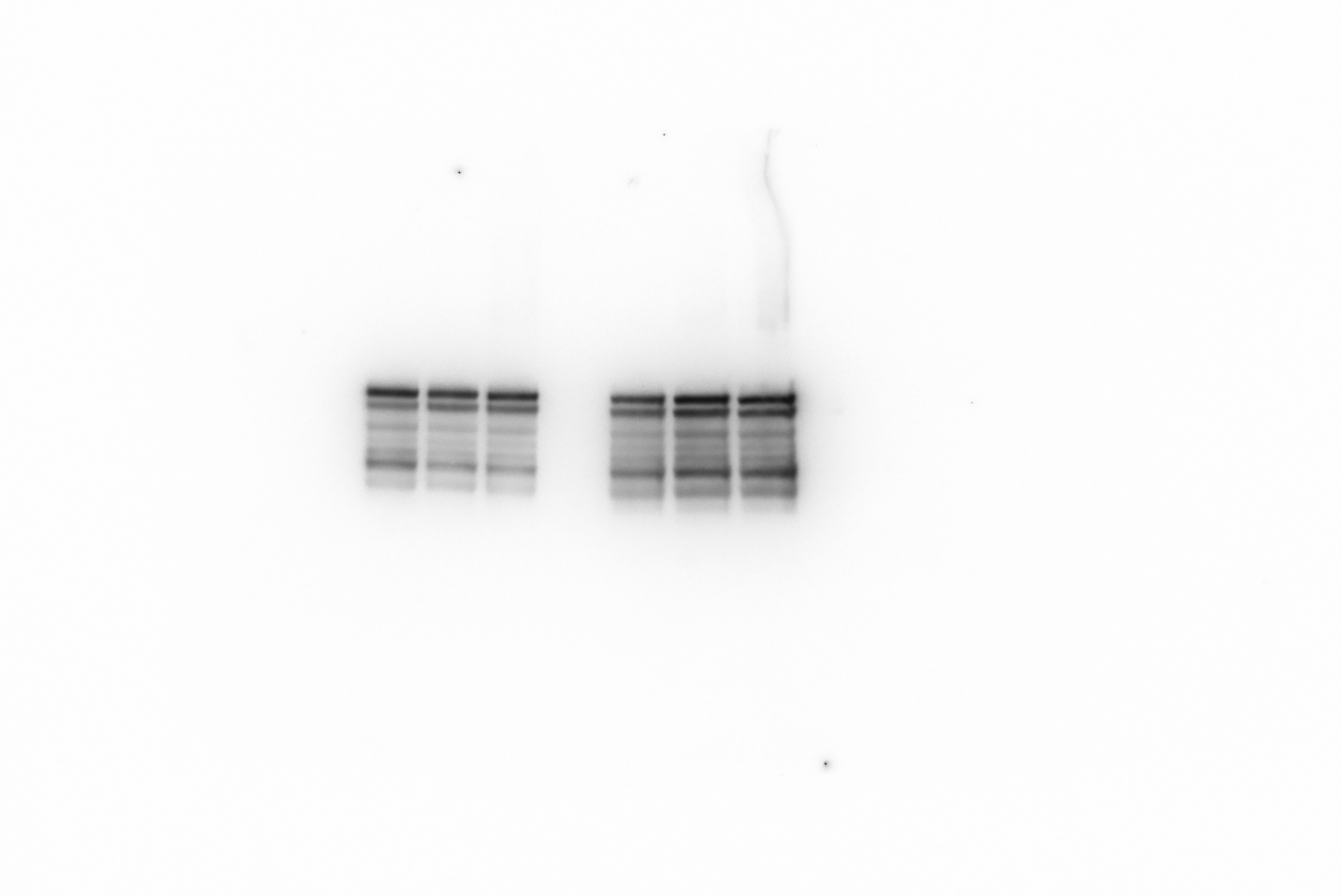

Supplement: Figure 5—source data 1. [file elife-72377-fig5-data1.zip › ECT2:3_Targets_v2_Figure5-Source_data_2.jpg]

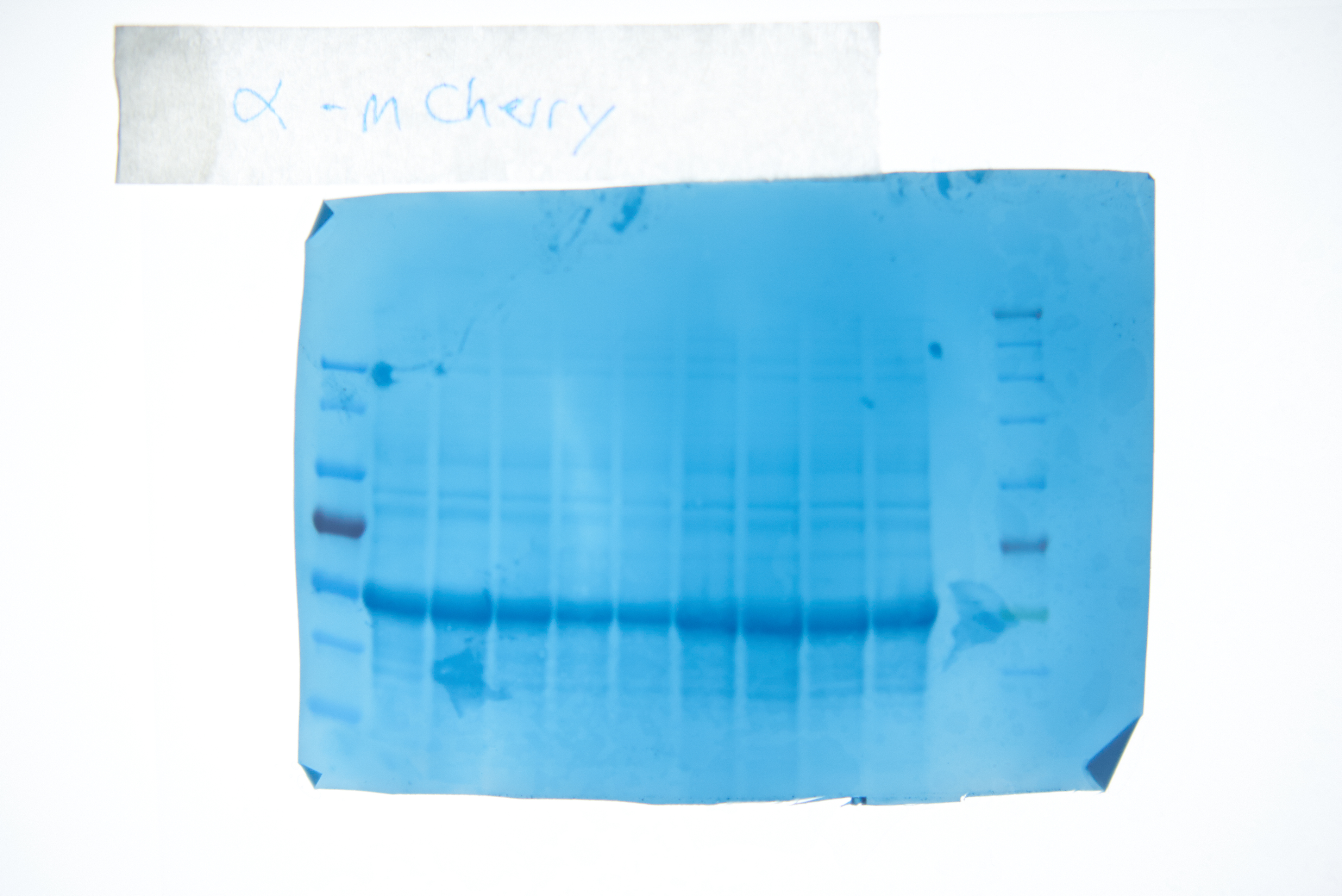

Supplement: Figure 5—source data 1. [file elife-72377-fig5-data1.zip › ECT2:3_Targets_v2_Figure5-Source_data_3.jpg]
